# Supplementary material for: Bayesian refinement of protein structures and ensembles against SAXS data using molecular dynamics
Source: PLoS Comput Biol. 2017 Oct 18;13(10):e1005800. doi: 10.1371/journal.pcbi.1005800 (PMC5662244; doi:10.1371/journal.pcbi.1005800)
Supplement: S1 Archive — (BZ2) [file pcbi.1005800.s011.tar.bz2 › gromacs_4.62_bayesian_saxs_refi/share/html/online/xtc.html]

xtc file format


## xtc file format

|  |  |
| --- | --- |
| Main Table of Contents | **VERSION 4.6** |
| GROMACS homepage | **Sat 19 Jan 2013** |

---

### Description

The xtc format is a **portable** format for trajectories.
It uses the *xdr* routines for writing and reading
data which was created for the Unix NFS system. The trajectories
are written using a reduced precision algorithm which works
in the following way: the coordinates (in nm) are multiplied by a scale
factor, typically 1000, so that you have coordinates in pm.
These are rounded to integer values. Then several other tricks are
performed, for instance making use of the fact that atoms close
in sequence are usually close in space too (e.g. a water molecule).
To this end, the *xdr* library is extended with a special routine
to write 3-D float coordinates. This routine was written by Frans van Hoesel
as part of an Europort project, and can be obtained through this link.

All the data is stored using calls to *xdr* routines.

**int** magic: A magic number, for the current file version its value is 1995. **int** natoms: The number of atoms in the trajectory. **int** step: The simulation step. **float** time: The simulation time. **float** box[3][3]: The computational box which is stored as a set of three basis vectors, to allow for triclinic PBC. For a rectangular box the box edges are stored on the diagonal of the matrix. **3dfcoord** x[natoms]: The coordinates themselves stored in reduced precision. Please note that when the number of atoms is smaller than 9 no reduced precision is used.

### Using xtc in your "C" programs

To read and write these files the following "C" routines are available:

```
/* All functions return 1 if successful, 0 otherwise */  

extern int open_xtc(XDR *xd,char *filename,char *mode);
/* Open a file for xdr I/O */
  
extern void close_xtc(XDR *xd);
/* Close the file for xdr I/O */
  
extern int read_first_xtc(XDR *xd,char *filename,
			  int *natoms,int *step,real *time,
			  matrix box,rvec **x,real *prec);
/* Open xtc file, read xtc file first time, allocate memory for x */

extern int read_next_xtc(XDR *xd,
			 int *natoms,int *step,real *time,
			 matrix box,rvec *x,real *prec);
/* Read subsequent frames */

extern int write_xtc(XDR *xd,
		     int natoms,int step,real time,
		     matrix box,rvec *x,real prec);
/* Write a frame to xtc file */
```

To use the library function include "xtcio.h"
in your file and link with -lgmx.$(CPU)

### Using xtc in your FORTRAN programs

To read and write these in a FORTRAN program use the calls to
readxtc and writextc as in the following sample program
which reads and xtc file and copies it to a new one:

```
      program testxtc
      
      parameter (maxatom=10000,maxx=3*maxatom)
      integer xd,xd2,natoms,step,ret,i
      real    time,box(9),x(maxx)
      
      call xdrfopen(xd,"test.xtc","r",ret)
      print *,'opened test.xtc, ret=',ret
      call xdrfopen(xd2,"testout.xtc","w",ret)
      print *,'opened testout.xtc, ret=',ret
      
 10   call readxtc(xd,natoms,step,time,box,x,prec,ret)
 
      if ( ret .eq. 1 ) then
         call writextc(xd2,natoms,step,time,box,x,prec,ret)
      else
         print *,'Error reading xtc'
      endif
         
      stop
      end
```

To link your program use -L$(GMXHOME)/lib/$(CPU) -lxtcf
on your linker command line.
The source for this test can be found in file
$(GMXHOME)/src/gmxlib/testxtcf.f.

---

http://www.gromacs.org
